# Hsp90 inhibitor HSP990 in very low dose upregulates EAAT2 and exerts potent antiepileptic activity

**DOI:** 10.7150/thno.44721

**Published:** 2020-07-09

**Authors:** Longze Sha, Ting Chen, Yu Deng, Tingfu Du, Kaili Ma, Wanwan Zhu, Yan Shen, Qi Xu

**Affiliations:** 1 State Key Laboratory of Medical Molecular Biology, Institute of Basic Medical Sciences Chinese Academy of Medical Sciences, School of Basic Medicine Peking Union Medical College, Beijing, 100005, China.; 2 Institute of Medical Biology, Chinese Academy of Medical Sciences and Peking Union Medical College, Kunming, 650118, China.; 3 Neuroscience center, Chinese Academy of Medical Sciences, Beijing, 100005, China.

**Keywords:** Temporal lobe epilepsy, Hsp90, EAAT2, HSP990

## Abstract

**Rationale:** Dysfunction or reduced levels of EAAT2 have been documented in epilepsy. We previously demonstrated the antiepileptic effects of Hsp90 inhibitor 17AAG in temporal lobe epilepsy by preventing EAAT2 degradation. Because of the potential toxicities of 17AAG, this study aimed to identify an alternative Hsp90 inhibitor with better performance on Hsp90 inhibition, improved blood-brain barrier penetration and minimal toxicity.

**Methods:** We used cell-based screening and animal models of epilepsy, including mouse models of epilepsy and Alzheimer's disease, and a cynomolgus monkey model of epilepsy, to evaluate the antiepileptic effects of new Hsp90 inhibitors.

**Results:** In both primary cultured astrocytes and normal mice, HSP990 enhanced EAAT2 levels at a lower dose than other Hsp90 inhibitors. In epileptic mice, administration of 0.1 mg/kg HSP990 led to upregulation of EAAT2 and inhibition of spontaneous seizures. Additionally, HSP990 inhibited seizures and improved cognitive functions in the APPswe/PS1dE9 transgenic model of Alzheimer's disease. In a cynomolgus monkey model of temporal lobe epilepsy, oral administration of low-dose HSP990 completely suppressed epileptiform discharges for up to 12 months, with no sign of hepatic and renal toxicity.

**Conclusions:** These results support further preclinical studies of HSP990 treatment for temporal lobe epilepsy.

## Introduction

Increasing evidence suggests that loss of glutamate transporter excitatory amino acid transporter 2 (EAAT2, also known as GLT-1) in astrocytes is responsible for neuronal hyperexcitability and spontaneous epileptic discharges in temporal lobe epilepsy (TLE) 1. We previously demonstrated that Hsp90 is upregulated in reactive astrocytes of human epileptogenic tissue and downregulates EAAT2 protein levels through proteasome-dependent degradation that could be prevented by the Hsp90 inhibitor 17AAG 2. Although long-term systemic administration of 17AAG largely inhibited epileptic seizures in a mouse model of TLE, its disadvantage is the potential for toxic effects 3. Additionally, because 17AAG does not efficiently cross the blood-brain barrier (BBB) 4, most of the administered 17AAG acts on the whole body, which leads to the development of adverse effects. It is thus necessary to find new inhibitors with improved efficacy of Hsp90 inhibition and low side-effects.

Using cell-based screening and animal models of epilepsy, we report here that low-dose HSP990 showed potent antiepileptic activity. In a cynomolgus model of TLE, long-term oral administration of HSP990 completely suppressed epileptiform discharges. No significant weight loss or abnormal blood indices were observed in monkeys after 12 months of treatment. The combination of these preclinical results with desirable pharmaceutical properties supports further evaluation of HSP990 in preclinical studies.

## Methods

### Reagents and chemicals

The following chemicals were used at the indicated concentrations: 17DMAG (MCE), STA-9090 (MCE), NMS-E973 (Selleck), BEP800 (MCE), HSP990 (Selleck), KA monohydrate (Sigma-Aldrich), and DMSO (Ameresco). The Hsp90 inhibitors were dissolved in PEG400/DMSO vehicle and administered directly into the stomach of mice via oral gavage. For the study of the antiepileptic activity of HSP990 in monkeys, each compound was dissolved in a vehicle comprising 90% PEG400 (Sigma-Aldrich) and 10% DMSO and filled into a hard gelatin capsule.

### Animals

Wild-type adult male or newborn C57BL/6J mice were obtained from Vital River. APP/PS1 transgenic mice (C57BL/6 mouse strain) were obtained from the Jackson Laboratory. The mice were housed in cages with littermates. To study the drug in the primate model of TLE, three young female and two young male cynomolgus monkeys (7-13 years old; 3.4-9.3 kg) were used in this study. Animals were handled in accordance with protocols approved by the Institutional Review Board of Chinese Academy of Medical Sciences and Peking Union Medical College and were conducted within the terms of the Beijing administration animal guidelines for the care and use of laboratory animals.

### Primary cultures of astrocytes

Cerebral cortices of newborn C57BL/6J pups were removed and cleaned of meninges and blood vessels. The remaining tissue was mechanically dissociated with a 25-gauge needle and suspended in DMEM (Macgene). Isolated cells were primary cultured on poly-d-lysine (PDL) coated T75 cm^2^ flasks (Falcon) in DMEM supplemented with 20% FBS (Gibco) and 10 ng/ml EGF (Peprotech), and maintained in a humid atmosphere of CO2/air (5%/95%) at 37°C for 5-7 d without changing the culture medium. After they reached confluence, microglia and oligodendrocytes were removed by placing the flasks in a heated shaker (37°C) overnight. Astrocytes were then replated onto PDL coated dishes for subsequent experiments. The Hsp90 inhibitors tested, including 17DMAG, Ganetespib, NMS-E973, BEP800 and HSP990, were obtained from Selleck.

### Quantitative real-time PCR

Total RNA from astrocyte cultures was extracted using Trizol (Thermo Fisher) according to the manufacturer's protocol. The cDNA was prepared using Fast Lane Cell cDNA Kit (Qiagen, CA, USA). Levels of mRNA were assessed by real-time PCR using SYBR supermix (Bio-Rad, CA, USA). A fragment of actin was amplified as the internal control. The following forward (F) and reverse (R) primers were used to amplify EAAT2 and actin: EAAT2 (F): 5-TAGATATGTCGGTTGCCGTTTG-3, EAAT2 (R): 5-AGCCCAGTCCACCAGTGAGG-3; Actin (F): 5-GAGATTACTGCCCTGGCTCCTA-3, Actin (R): 5-TCATCGTACTCCTGCTTGCTGAT-3. Differences in gene expression were calculated using the 2-deltaCT method and are presented as the relative fold change.

### Western blot analysis

Protein extraction and Western blotting were performed as previously described 5. A 10 μg portion of protein from whole cell lysates or 20 μg of protein from tissue lysates was run on SDS-PAGE gels, blotted onto nitrocellulose and blocked in TBST + 5% nonfat dry milk. The following antibodies were used: including those against Hsp90α (#109248) and Hsp90β (#53497) from Abcam, those against Actin (#20536), Hsp90 (#13171), Hsp70 (#10995) and EAAT2 (#22515) from Proteintech as well as the antibody against EAAT2 (#15317, Santa Cruz Biotechnology).

### Anatomical processing, immunohistochemistry (IHC), and analysis procedures

The following primary antibodies were used: including those for GFAP, Hsp90, Hsp70 and beta-amyloid (6E10). Photographs were captured with an inverted fluorescence microscope (DMI4000B; Leica) or confocal laser scanning microscope (880; Zeiss). Amplifier offset and detector gain were adjusted first and never changed in the course of an experimental session. Comparison of Hsp90 IF intensity between neurons and astrocytes was performed using ImageJ software. The Hsp90 IF intensity was measured from 60 astrocytes in the sclerotic hippocampus and 60 neuronal cells equally selected from the hippocampus, cortex and thalamus, respectively. Three images from a series of sections for each mouse were collected. To quantify Hsp70-positive astrocytes, three coronal sections along the anterior-posterior axes of the hippocampus of each mouse were used for Hsp70 and GFAP immunostaining and quantification. The number of Hsp70^+^/GFAP^+^ astrocytes in the hippocampus was manually counted. The images of Aβ plaque (6E10) immunoreactivity were analyzed with Image J software. The mean of these five images (20× magnification) was calculated to represent the averaged 6E10-labeled area in the hippocampus of each individual mouse. The data are represented as a percentage of the 6E10-labeled area out of the total areas.

### Hippocampal microdialysis

*In vivo* microdialysis and glutamate concentration assays were performed as previously described 6.

### Hippocampal metabolomics

At the endpoint of vEEG monitoring, the mice were euthanized for metabolomics profiling. An aliquot of each individual hippocampus was precisely weighed and transferred to an Eppendorf tube. After the addition of 500 μL of extract solvent (precooled at -20°C, acetonitrile/methanol/water, 2:2:1), the samples were vortexed for 30 s, homogenized at 45 Hz for 4 min using a TissueLyser LT, and sonicated for 5 min in an ice-water bath. The homogenizing-sonicating cycle was repeated 3 times, followed by incubation at -20°C for 1 h and centrifugation at 12,000 rpm and 4°C for 15 min. A 100 μL aliquot of the clear supernatant was transferred to an auto-sampler vial for UHPLC-MS/MS analysis (Agilent).

### Acute and chronic model of PTZ-induced seizures

Pentylenetetrazole (PTZ, Sigma-Aldrich) was dissolved in 0.9% saline, filter sterilized, and administered intraperitoneally in a volume of 0.2mL/0.03 kg. Male C57BL6 mice received HSP990 three times at 0.5 mg/kg once every other day via oral gavage. Twenty-four hours after mice received the last HSP990 treatment, acute seizures were induced by a single dose of 55 mg/kg PTZ**.** Dihydrokainic acid (DHK, Santa Cruz) was used as an EAAT2 inhibitor to block the effects of HSP990, which was intraperitoneally injected 30 min before PTZ.

To study the effects of HSP990 on epileptogenesis, mice were repetitively injected with 40 mg/kg PTZ once every other day. HSP990 was administrated on the day before each PTZ injection. Seizures score was graded by an observer blinded to the experimental condition using a 5 point seizure score: 0) no behavioral signs; 1) whisker trembling, and/or facial jerking, neck jerks; 2) clonic seizure in a sitting position; 3) tonic-clonic seizure (lying on belly); 4) tonic-clonic seizure (lying on side) or wild jumping.

### Kainic acid (KA) model of chronic TLE and video-EEG (vEEG) analysis

The KA-induced mouse model of chronic TLE, wired vEEG recording and statistical analysis were established as previously described 7. Four or five weeks after unilateral hippocampal injection of KA, mice exhibit chronic spontaneous seizures. The baseline of motor seizures was measured for 14 consecutive days before vehicle or HSP990 administration, and mice with seizures at least 4 times per week were chosen for follow-up study. The selected mice were randomly divided into 2 groups, including 10 mice in the vehicle group and 14 mice in the HSP990 group. There was no difference in the baseline of spontaneous seizures between the vehicle and HSP990 groups. HSP990 or vehicle was delivered by oral gavage on a schedule of every other day. During vehicle or HSP990 administration, two mice in the HSP990 group and three mice in the vehicle group died of lethal seizures. At the end of the experiment, vEEG data were analyzed in seven vehicle-treated mice and 12 HSP990-treated mice, respectively. The baseline seizure frequency (number of seizures per day) was measured from 14-days vEEG data recorded before vehicle or HSP990 administration. Seizure frequency during vehicle or HSP990 treatment was measured from vEEG data acquired between the day 1 and the endpoint. For each animal, we attempted to record their EEG as long as possible during HSP990 or vehicle administration (up to 80 days in this study), however, as the EEG head implant might become loose contact between EEG electrode and scalp in those free-moving mice, we terminated EEG recording and treatment when the EEG signal became noisy. Finally, there was no significant difference between the mean treatment days in the vehicle and HSP990 group were 38 (min to max: 20 - 50) days and 45 (min to max: 20 - 81) days, respectively (p = 0.4 by Student's t test).

### Morris water maze

The Morris water maze test was performed as previously described 8. During the hidden-platform test, the mice were given 2 trials per day. During each trial, the mice were released from four pseudo-randomly assigned starting points and allowed to swim for 60 s. After mounting the platform, the animals were allowed to remain there for 10 s and were then placed in the home cage until the start of the next trial. If a mouse was unable to find the platform within 60 s, it was guided to the platform and allowed to rest on the platform for 10 s. Probe trials were performed on day 10. In the probe trial, the hidden platform was removed, and the animal was released from the right quadrant and allowed to swim freely for 45 s. The time spent in the target zone, where the platform had been located during training, was measured. All experiments were conducted at approximately the same time each day. For the hidden-platform test, the data of two trials per day for each mouse were averaged, and the differences in escape latency between groups were analyzed. For the visible-platform test, the data of four trials from 2 days were compared between groups. For the probe test, the data of 2 two trials for each mouse were averaged, and the time spent in the target zone was compared between groups.

### The cynomolgus monkey model of TLE and drug treatment

Five cynomolgus monkeys were used to establish a TLE model as previously described 9. The monkeys were divided into two groups, including three monkeys in the HSP990 group that received oral administration with a hard gelatin capsule comprising HSP990 in 100 μL of PEG400/DMSO solvent (volume ratio 9:1) mixed with starch (0.5 g) and two monkeys in the vehicle group that received the same hard gelatin capsule comprising an equal volume of PEG400/DMSO solvent and starch without HSP990. These five monkeys were treated on a schedule of every other day for up to 12 months.

### EEG recording for cynomolgus monkeys

Because no obvious convulsive symptoms were observed in the unilateral hippocampal KA model of macaque monkeys after KA injection 9-11, the frequency of epileptic spikes was measured in each monkey to evaluate the antiepileptic activity of HSP990. EEG was recorded using a Cyton Biosensing Board wireless module with a sampling rate of 250 Hz (OPENBCI). After sodium pentobarbital-induced anesthesia, the animals were restrained in a primate chair, and scalp EEG recordings were performed for at least 1.5 h before the monkeys were recovered from anesthesia (temporal cortex T3/T4, frontal cortex F3/F4, and Cz reference). Acquired EEG data were converted into the EEG file format and processed using EEGLAB software. The bandpass filter for the EEG data was set from 1 to 50 Hz. For a transient to be deemed as an interictal epileptic discharge, several previously described criteria had to be fulfilled. Generally, interictal spikes were identified and defined as fast epileptiform waveforms that had a peak amplitude 2-fold greater than the average background EEG and a characteristic spike component lasting between 30 and 50 ms. For the analysis, the sum of epileptic spikes was manually counted in the second 30-min segment of signal recorded.

### Pharmacokinetics analysis and safety evaluation

Pharmacokinetic samples were obtained and evaluated in the HSP990-treated monkeys to characterize the disposition of HSP990 after oral administration at various dose and post-dosing time points (1 h, 24 h and 48 h). The concentrations of HSP990 in plasma were determined by isocratic reversed-phase HPLC with electrospray ionization mass spectrometric (LC/MS-MS) detection. The method showed a lower limit of quantification (LLOQ) of 50 pg/mL, The quality control (QC) samples were prepared in blank plasma at the concentrations of 50, 100, 250, 1250, 6250, 12500, 20000, and 25000 pg/mL, respectively. For each validation, six replicates were analyzed for each QC level (three validations were performed). The precision and accuracy of assay was determined at four different concentrations (50, 150, 7500, and 18750 pg/mL). The following criteria were found to approve precision and intra-race accuracy: (i) for each concentration level, coefficient of variation (CV) that does not exceed 15% for LLOQ and 15% for QC samples; (ii) mean value of the samples at each concentration level, within 85 - 115% of the actual value for LLOQ and 80-120% for QC samples.

Safety assessment consisted of blood tests and weight. Blood tests, including haematology parameters (n = 15) and serum biochemistry parameters (n = 14), were carried out on an automatic blood-counter system. Examinations were conducted at baseline (before treatment), during treatment and at the end point of the experiment. The changes in key parameters were used to assess potential hepatic toxicity (AST/ALT) and renal toxicity (BUN/CREA) before and after oral administration of HSP990.

### Statistics

Statistical significance was examined by two-tailed tests using Prism software (GraphPad), such as Student's t-test, Mann Whitney test, or paired Student's t-test. One-way ANOVA or Two-way ANOVA was used for multiple comparisons. The data were presented as the mean ± SD unless otherwise noted. All EEG analyses were performed in a blinded manner.

### Data availability

The authors confirm that the data supporting the findings of this study are available within the article and/or its Supplementary material.

## Results

### Screening of new Hsp90 Inhibitors

**Figure 1:**
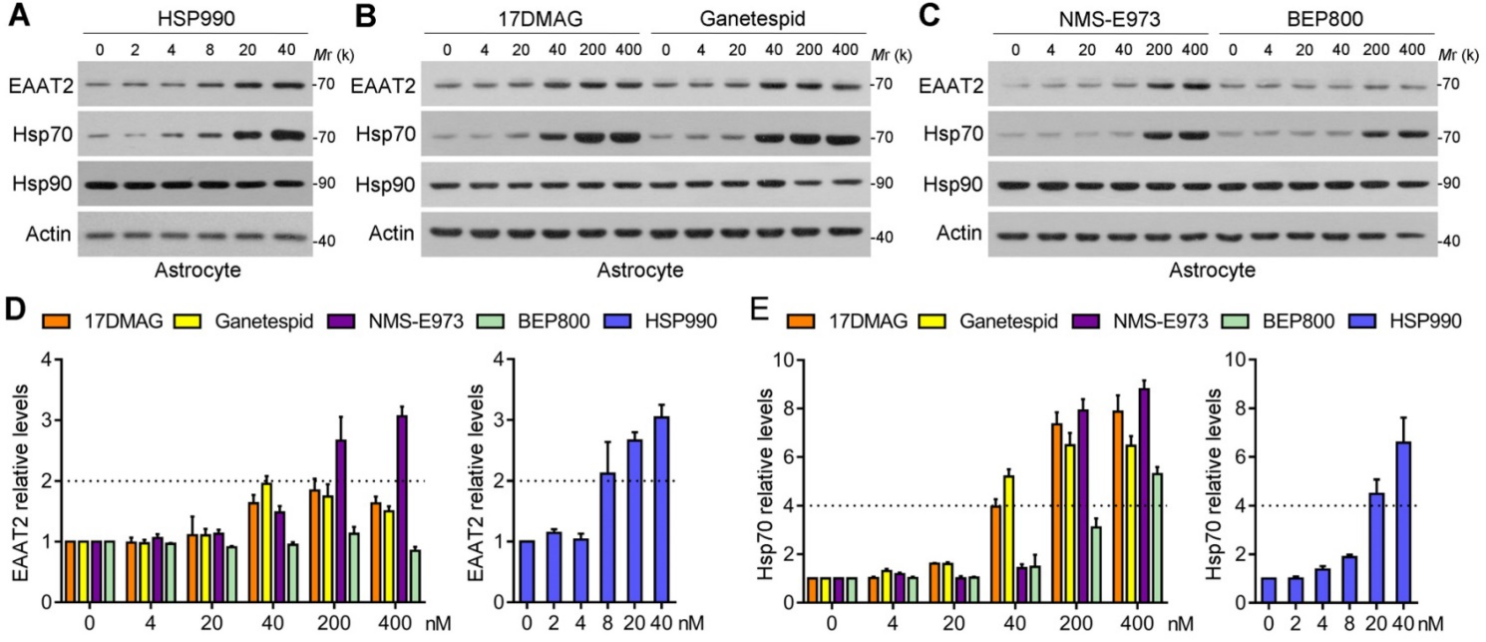
** Screening of the 5 Hsp90 inhibitors in primary cultured astrocytes. (A-C)** Western blot analysis of three proteins of interest in astrocytes treated with the 5 Hsp90 inhibitors for 24 h. **(D)** Dose-dependent changes of EAAT2 protein levels after 24-h treatment with 5 Hsp90 inhibitors, and **(E)** Dose-dependent changes of Hsp70 protein levels after 24-h treatment with 5 Hsp90 inhibitors. n = 3 for each group. Data are representative of two independent experiments.

Five representative new-generation Hsp90 inhibitors were selected for tests including 17DMAG, HSP990, NMS-E973, Ganetespib and BEP800. Hsp90 inhibitors generally lead to significant activation of HSF1 and subsequent upregulation of Hsp70; therefore, Hsp70 was examined as a biomarker for Hsp90 inhibition. We first evaluated their efficacy of inducing EAAT2 levels in primary cultured astrocytes. Similar to 17AAG, 4 Hsp90 inhibitors selected could increase EAAT2 levels in a dose-dependent manner, in which HSP990 treatment upregulated EAAT2 protein levels by 2.1-fold at 8 nM concentration (Figure 1A). To achieve the same effect, however, the concentrations of Ganetespid, 17DMAG and NMS-E973 had to reach 40-200 nM (Figure 1B-C). Although BEP800 markedly induced Hsp70 upregulation at 200 or 400 nM, EAAT2 was not affected for unknown reasons (Figure 1D-E). None of the five compounds affects the levels of EAAT1, another critical glutamate transporter in astrocytes (Figure S1).

**Figure 2:**
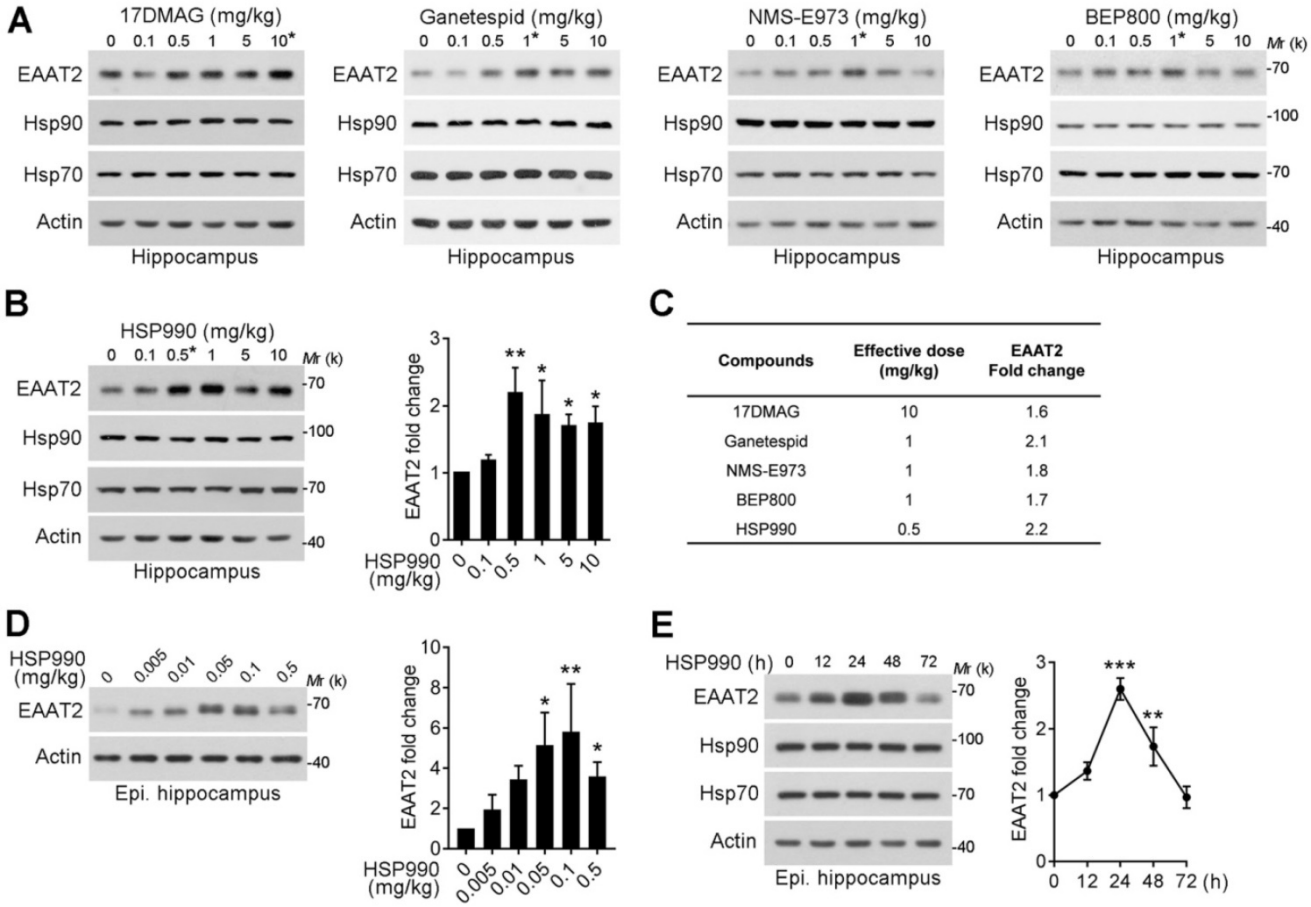
** Upregulation of EAAT2 levels by low-dose HSP990 treatment in normal and epileptic mice. (A, B)** Representative Western blot of EAAT2 in the hippocampus of normal mice after oral administration of the 17DMAG, Ganetespid, NMS-E973, BEP800 and HSP990 three times within a week (n = 3 for each dose).** (C)** The dose required for each of the 5 Hsp90 inhibitors to maximally increase EAAT2 levels in the hippocampus of normal mice. **(D)** Representative Western blot of EAAT2 in the hippocampus of epileptic mice after oral administration of HSP990 three times within a week; bar graph shows the fold change in EAAT2 levels (n = 3 for each dose). **(E)** Western blot of EAAT2 levels in the hippocampus from HSP990-treated epileptic mice at the indicated time point after HSP990 administration (0.1 mg/kg); bar graph shows the fold change in EAAT2 levels (n = 3 for each time point). * P < 0.05; ** P < 0.01; *** P < 0.01, one-way ANOVA with post-hoc Dunnett's test.

Next, the compounds were given to normal mice and KA model of chronic TLE by oral gavage three times within a week, and the hippocampi were then dissected for western blot analysis. As shown in Figure 2A-B, the effective dose to induce EAAT2 upregulation differed: HSP990 increased EAAT2 protein levels at a much lower dose than the other four inhibitors in normal mice (0.5 mg/kg; Figure 2A-C). Interestingly, the optimal dose of 0.1 mg/kg HSP990 to increase EAAT2 levels in epileptic mice was five times lower than in normal mice (Figure 2D). We speculated that this might be because of the compromised BBB of the sclerotic hippocampus, as reported before 12. In epileptic mice, the highest dose of HSP990, 0.5 mg/kg, resulted in a lower EAAT2 increase than the dose of 0.1 mg/kg (Figure 2B and D), which might be attributed to inhibited EAAT2 mRNA levels induced by toxic effects of high-dose (Figure S2).

Additionally, we examined the time course of the effects of HSP990 on EAAT2 levels in epileptic mice. The EAAT2 levels peaked at 24 h oral administration and began to decrease thereafter, but were still 1.7-fold higher than the basal level at 48 h (Figure 2E). In summary, low-dose HSP990 can effectively induce an increase of hippocampal EAAT2 levels in epileptic mice.

### Hsp90 expression in Astrocytes is lower than in Neurons

**Figure 3:**
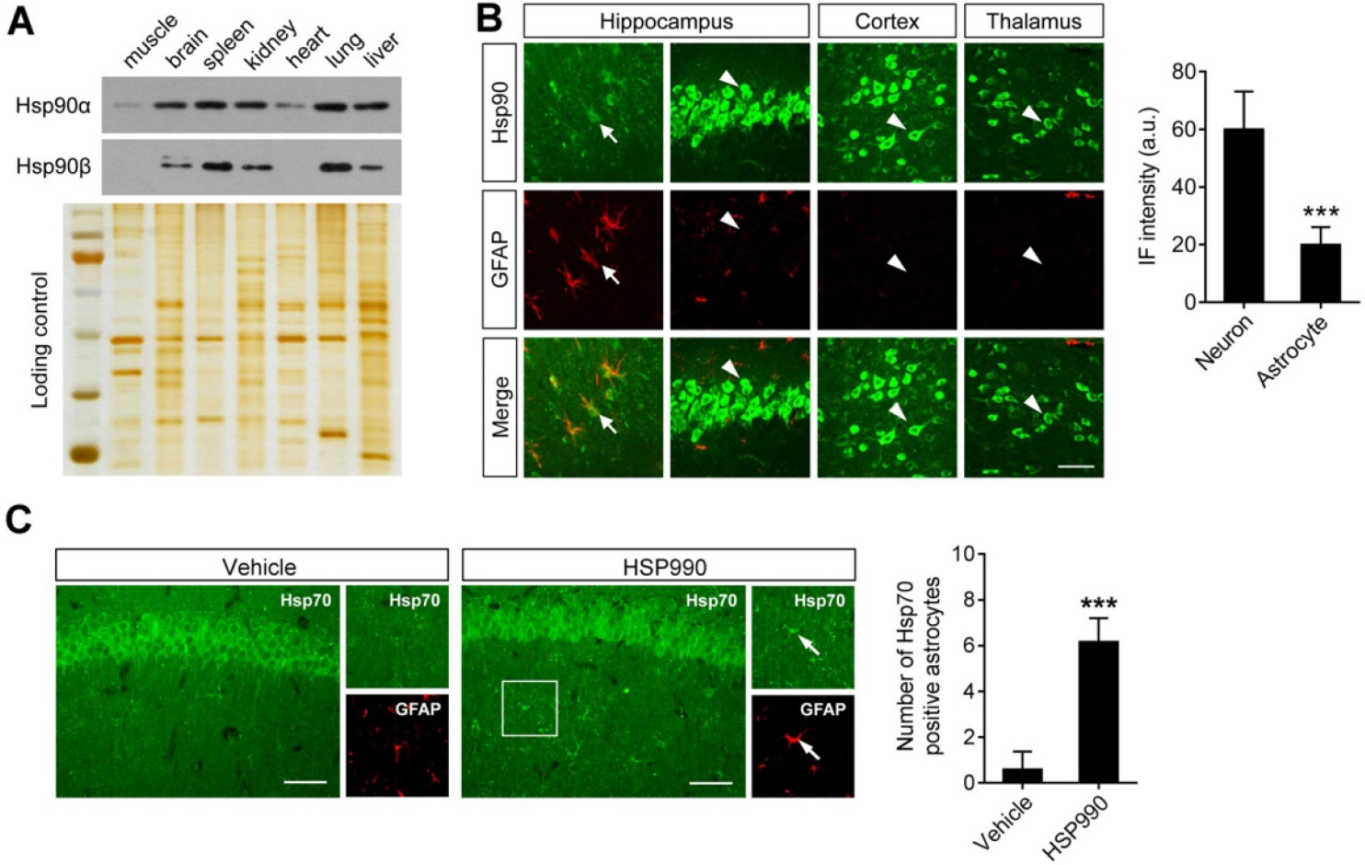
** Differential expression of Hsp90 in astrocytes and neurons. (A)** Western blot of Hsp90α and Hsp90β to show protein expression in different tissues from epileptic mice (including muscle, brain, spleen, kidney, heart, lung and liver). Silver-stained gel was used as a loading control. **(B)** Representative images of the hippocampus, cortex and thalamus from epileptic mice immunostained with Hsp90 (green) and with an astrocyte-specific antibody GFAP (red). Scale bars: 50 µm. Student's t-test showed a significant difference in immunofluorescence intensity of Hsp90 per astrocyte and neuron (right; n = 3 mice per group; Student's t-test).** (C)** Representative images of the hippocampus immunostained with Hsp70 at 12 h of HSP990 administration (0.5 mg/kg). Scale bars: 100 µm. Quantification of Hsp70^+^/GFAP^+^ cells was performed (right; n = 4 mice per group; Student's t-test). a.u., arbitrary units. *** P < 0.001.

The above study demonstrated that a dose of 0.1 mg/kg was sufficient to upregulate EAAT2 levels. Although Hsp70 upregulation is a hallmark of Hsp90 inhibition, the western blot analysis failed to show a change in Hsp70 levels in the hippocampus after HSP990 treatment (Figure 2D). To explain the mechanism by which such a low-dose of HSP990 works, we investigated the expression of both Hsp90 and Hsp70 at a cellular level. Overall Hsp90 expression in brain homogenates was similar to that in the spleen, kidney, and lung (Figure 3A). Immunofluorescence (IF) analysis with an antibody against total Hsp90 demonstrated that Hsp90 staining in GFAP-positive astrocytes (arrows) in the brain of epileptic mice was much dimmer than in neurons (arrow heads), and the IF intensity was reduced by 67% in astrocytes compared with neurons (Figure 3B). The differences in cell type-specific expression of Hsp90 raise the possibility that a low concentration of Hsp90 inhibitor could suppress astrocytic Hsp90 with minimal effect on neuronal Hsp90. Indeed, we observed more Hsp70-positive astrocytes in the HSP990-treated hippocampus than in the vehicle-treated group, while the expression of Hsp70 in neuronal cells was not affected (Figure 3C). Considering that EAAT2 is predominantly expressed in astrocytes and inhibition of Hsp90 in astrocytes leads to increased EAAT2 levels, the advantage of a lower Hsp90 expression in astrocytes than in neurons offers a chance to reduce the dose of systemic administration of HSP990 and therefore alleviate side effects.

### HSP990 Reduces Seizures in Acute and Chronic Models of Epilepsy

**Figure 4:**
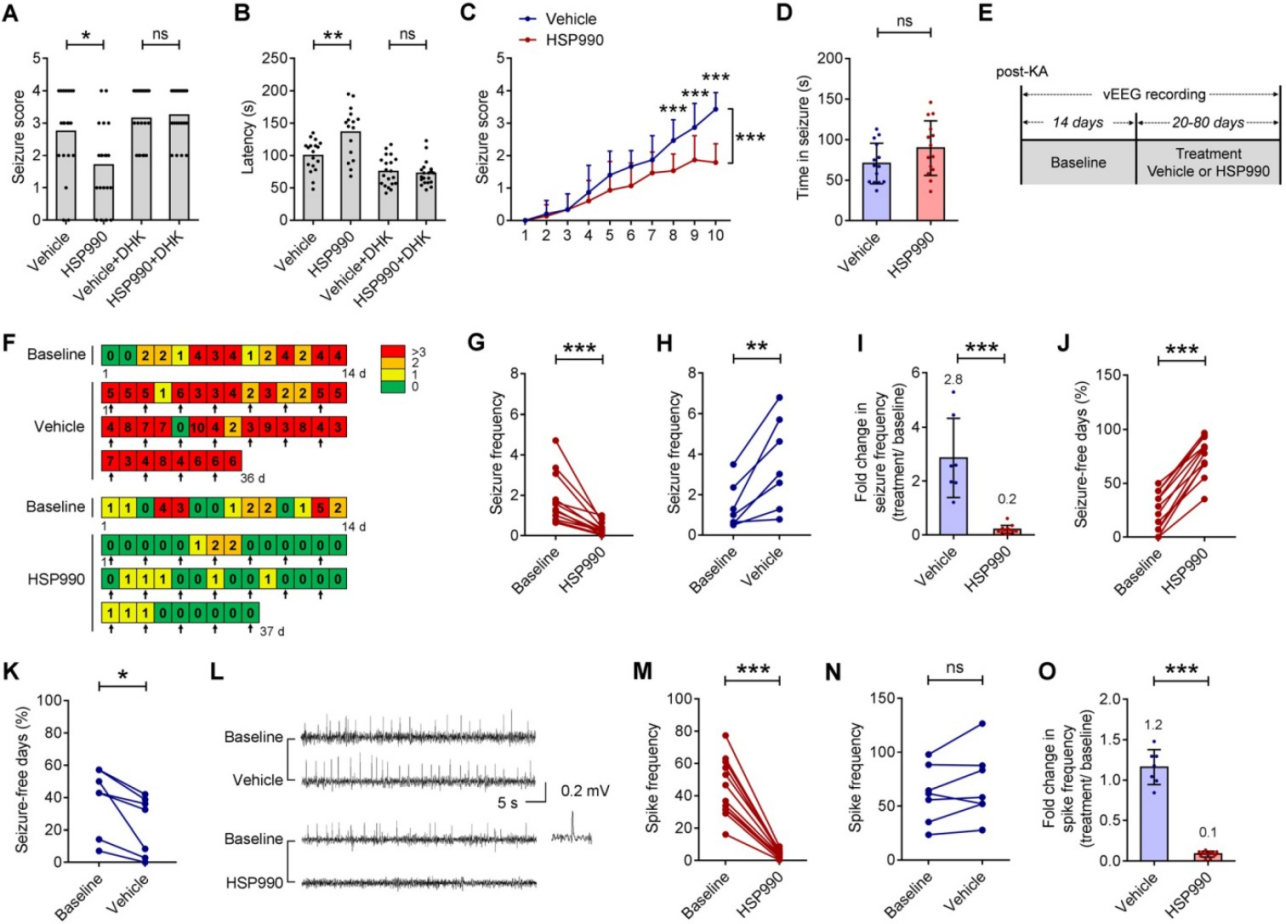
** The antiepileptic effect of HSP990 in acute and chronic models of epilepsy. (A, B)** Acute model of epilepsy. Mann Whitney test for the difference in seizure score and Student's t-test for latency of the acute response to PTZ.** (C, D)** PTZ kindling model of epilepsy. **(C)** Two-way ANOVA and post-hoc multiple analysis for the difference in seizure score. **(D)** Student's t test for duration of seizure after the last PTZ injection.** (E)** The experimental design of TLE animal model and drug administration. **(F)** Examples of daily recording of spontaneous recurrent seizures in the vehicle and HSP990 groups. **(G, H)** Paired t-test for the difference in the seizure frequency (average number of seizures per day) between baseline and treatment. **(I)** Student's t-test for the difference in fold change in seizure frequency before and after treatment with either vehicle or HSP990.** (J, K)** Paired t-test for the differences in percentage of seizure-free days between baseline and treatment. **(L)** Representative 1-min EEG tracings in baseline and recorded during treatment with HSP990 or vehicle, respectively. **(M, N)** Paired t-test for the difference in interictal epileptic spikes between the vehicle group and the HSP990 group.** (O)** Mann Whitney test for the difference in fold change of interictal epileptic spikes before and after treatments with either vehicle or HSP990. * P < 0.05; ** P < 0.01; *** P < 0.001. ns, not significant.

Given the role of HSP990 in increasing EAAT2 protein levels, we applied pharmacological interference to an acute model of epilepsy induced by a single dose of 55 mg/kg pentylenetetrazol (PTZ). Three-time oral administration of HSP990 at 0.5 mg/kg prior to PTZ administration decreased the severity of seizures in mice (Figure 4A). The percentage of animals in both groups responding to 55 mg/kg PTZ with a convulsion (18/20 and 17/20 of animals administrated with the vehicle or HSP990, respectively) was not different (Fisher's exact test, p = 0.66). The average seizure score in response to a 55 mg/kg PTZ challenge in HSP990 groups was lower than those in the control group (Figure 4A). HSP990-treated mice showed a delayed latency of the response to PTZ (Figure 4B). DHK, an EAAT2 specific inhibitor, completely blocked the effects of HSP990 (Figure 4A and B). To examine whether HSP990 affects epileptogenesis, mice were repetitively injected with 40 mg/kg PTZ. In the vehicle group, PTZ kindling increased seizure severity after 10 PTZ injections (Figure 4C), while simultaneous treatment with HSP990 decreased the average seizure score, but did not affect seizure duration (Figure 4D).

We next examined whether low-dose HSP990 can decrease spontaneous seizures in a mouse model of TLE. Figure 4E-F illustrates the experimental procedures, whereby arrows in Figure 4F indicate vehicle or HSP990 administration. Using an intra-hippocampal KA model of TLE, we found that the administration of HSP990 dramatically suppressed epileptic seizures during treatment (Figure 4G), an average decrease of 80% compared with baseline (Figure 4I). Conversely, the seizure frequency in the vehicle group had a 2.8-fold increase after vehicle treatment (Figure 4H-I). The average percentage of seizure-free days at baseline was 24%; this increased to 76% during HSP990 treatment (Figure 4J). Conversely the average percentage of seizure-free days in the vehicle group was decreased from 40% to 23% (Figure 4K). HSP990 treatment also substantially decreased the interictal epileptiform discharges (termed epileptic spikes), which was not observed in the vehicle group (Figure 4L-O). Taken together, our data suggest that low-dose HSP990 exhibits an antiepileptic effect in mice with chronic spontaneous epilepsy.

### The HSP990 Modulates Extracellular and Intracellular Glutamate Levels

**Figure 5:**
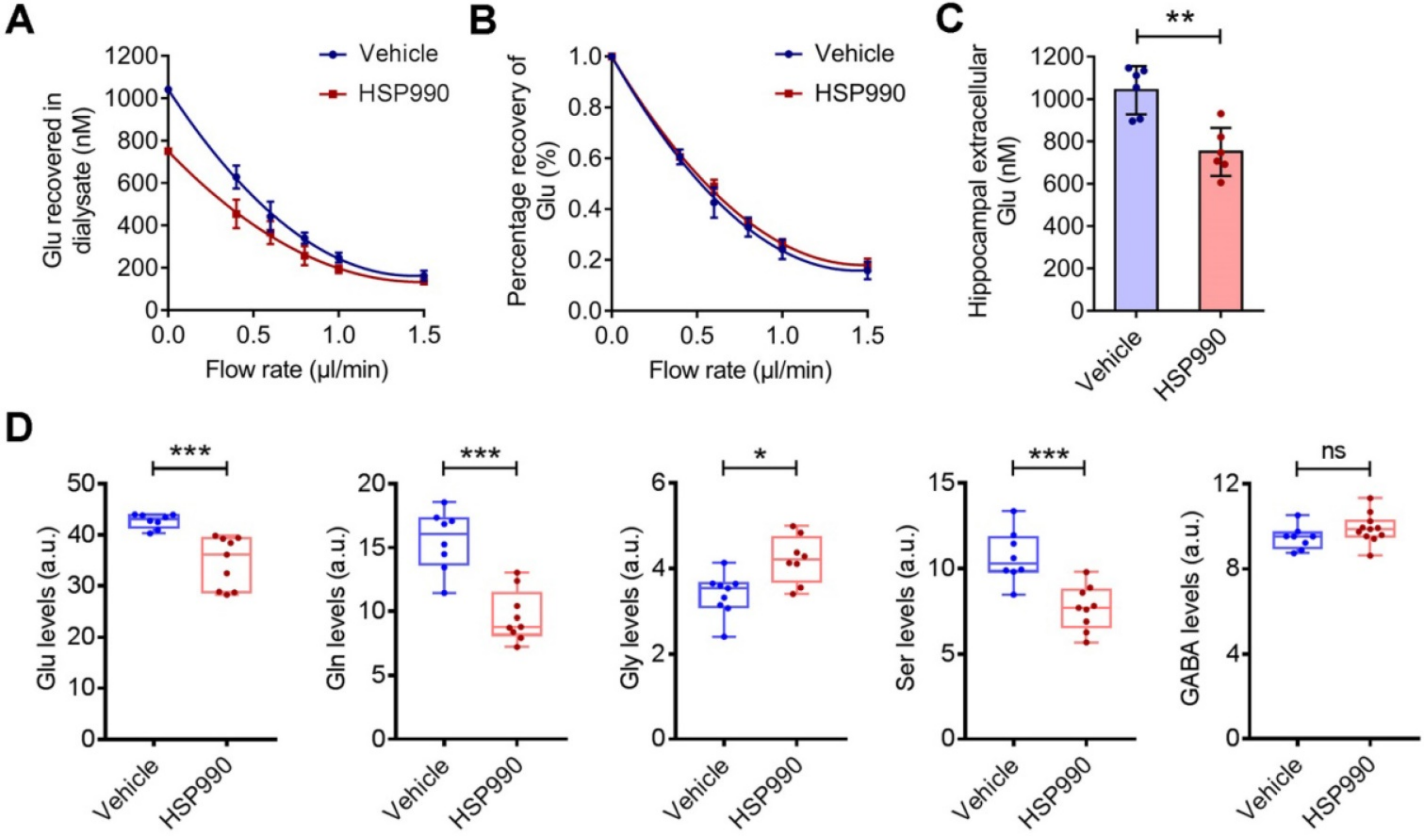
** The effects of HSP990 on extracellular and intracellular glutamate levels. (A)** Extracellular glutamate concentrations in the vehicle and HSP990 groups were measured by the extrapolated zero-flow method.** (B)** The percent recovery of glutamate at various flow rates was not significantly different between the vehicle group and the HSP990 group. **(C)** Mann Whitney test for the difference in hippocampal extracellular glutamate levels between the two groups (n = 6 each group,). **(D)** Mann Whitney test showed a significant difference in the levels of glutamate, glutamine, glycine, serine and γ-aminobutyric acid (GABA) in the hippocampus between vehicle-treated and HSP990-treated epileptic mice (n = 8-9 per group). * P < 0.05; ** P < 0.01; *** P < 0.001. a.u., arbitrary units.

EAAT2 removes glutamate from the extracellular spaces and thereby protects neurons against harmful activation of glutamate receptors. We examined hippocampal interstitial glutamate levels by microdialysis in epileptic mice treated with HSP990 and vehicle, respectively, for 3 weeks. As expected, we observed a significant reduction in the glutamate concentration in the HSP990 group (0.75 ± 0.11 μM) compared with the vehicle group (1.04 ± 0.11 μM) (Figure 5A-C). Metabolomic profiling showed that both glutamate and glutamine levels were decreased in the hippocampus of mice treated with HSP990 compared with the vehicle group (Figure 5D). Additionally, significant alterations in glycine and serine levels, which are co-agonists at NMDA receptors, were observed. Conversely, GABA and the other 15 amino acids were not changed (Figure 5D and Figure S3). These results suggest that HSP990 effectively promotes glutamate clearance from extracellular spaces and, at least in the hippocampus, modulates the metabolism of glutamate and other key amino acids.

### HSP990 Restores EAAT2 Levels, Inhibits Seizures and Improves Cognitive Functions in Mouse Model of Alzheimer's disease (AD)

**Figure 6:**
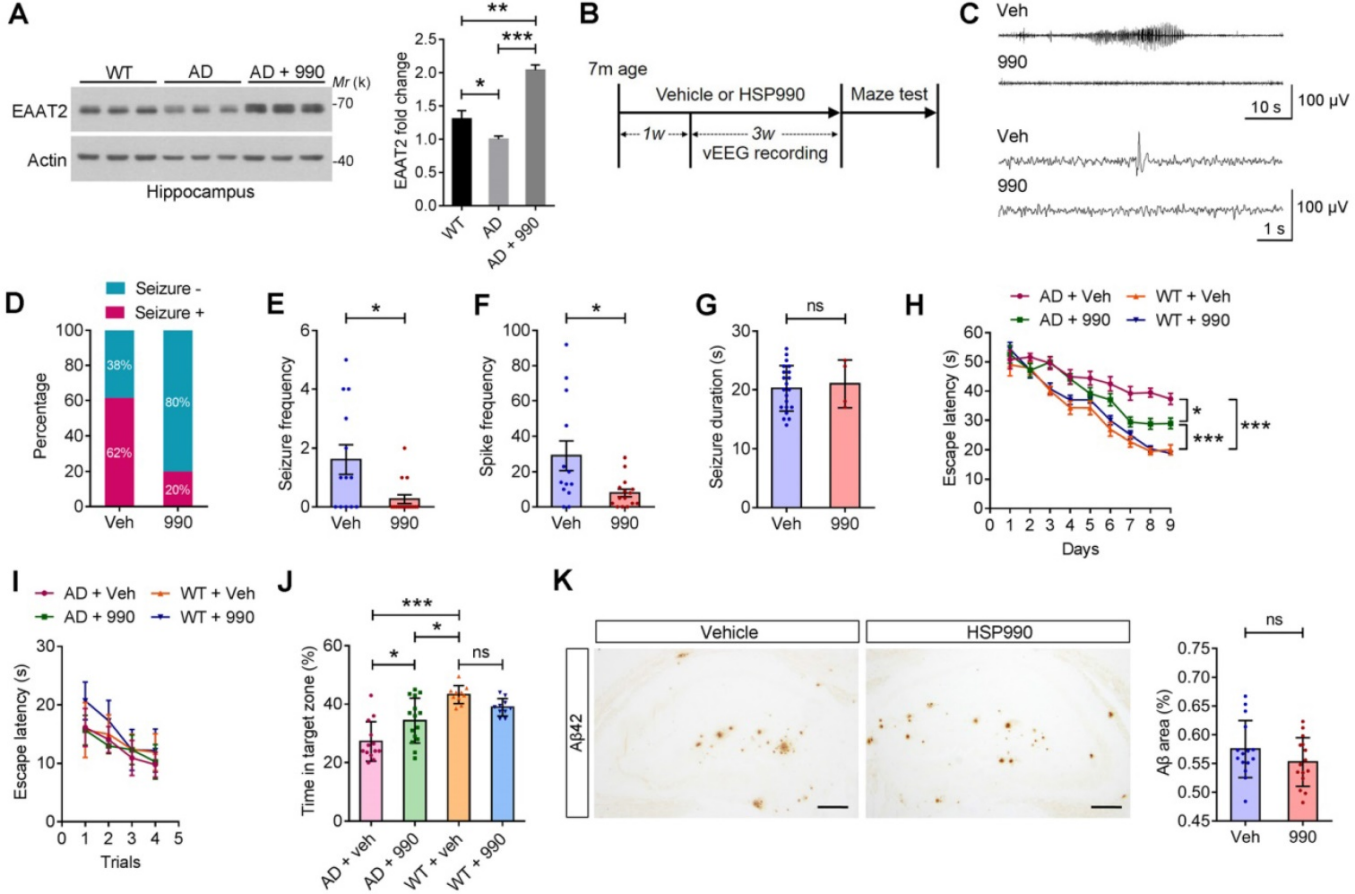
** Protective effects of HSP990 in the APP/PS1 mouse model of AD. (A)** Western blot of EAAT2 in the hippocampus of WT and AD mice after receiving oral administration of either vehicle or HSP990 three times within a week. Bar graph shows the fold change in EAAT2 (n = 3 mice per group). **(B)** Experimental design of the antiepileptic study and behavioral test. **(C)** Representative 1 min (up) or 10 s (down) EEG tracings recorded from mice treated with vehicle and HSP990, respectively. **(D)** The percentage of mice with at least one seizure in all mice. **(E-G)** The overall frequencies of seizures in the 3 weeks of EEG recording, the average frequency of spikes per hour, and the average seizure duration (vehicle, n = 13; HSP990, n = 15). **(H-J)** Morris water maze test. Escape latencies in the hidden-platform, visible-platform, and probe tests, respectively (vehicle, n = 13; HSP990, n = 15). 'Target zone' indicates the area where the platform was constantly located during the hidden-platform test. **(K)** Representative images of Aβ staining in the dentate gyrus region are shown, and quantitative analysis of Aβ staining (n = 4-5 mice each group). Veh, vehicle; 990, HSP990; ns, not significant. Student's t-test was used for all statistical analysis in this figure. * P < 0.05; ** P < 0.01; *** P < 0.001.

Epilepsy is a common disease phenotype observed in the APPswe/PS1dE9 transgenic model of AD. Loss of EAAT2 may be responsible for the aberrant neuronal discharge 13. Accordingly, we investigated whether HSP990 could restore EAAT2 levels in AD mice. The EAAT2 levels were reduced in the AD mice aged 7 months compared with normal mice, and administration of HSP990 restored EAAT2 levels (Figure 6A). To determine whether HSP990-induced EAAT2 has antiepileptic effects, 7-month old AD mice were treated with HSP990 or vehicle for 4 weeks (Figure 6B-C); their EEG was analyzed from the second week to the fourth week. In the vehicle group, 62% of the mice exhibited at least one seizure during the 3-week EEG recording, compared with 20% in the HSP990 group (Figure 6D). The overall frequencies of seizures and spikes were significantly decreased in the HSP990 group compared with the vehicle group (Figure 6E-F). No difference was observed in the time of each ictal episode (seizure duration) between the HSP990 group and the vehicle group (Figure 6J).

After 4 weeks of treatment, the Morris water maze test was used to measure spatial and learning memory. Mice in all groups readily found the cued platform (Figure 6I). Hidden-platform training revealed that HSP990 treatment rescued spatial learning impairment in the AD group, as demonstrated by significantly shorter escape latencies (Figure 6H). A probe trial was conducted 24 h after the last hidden-platform session to assess memory for the former platform location. The HSP990-treated AD mice spent significantly more time in the target zone compared with vehicle-treated AD mice (Figure 6J). Although HSP990 improved cognitive function in AD mice, it did not lower plaque burden in the hippocampus (Figure 6K), which suggests that the cognitive improvement is independent of amyloid deposition.

### Low-dose HSP990 Inhibits Epileptic Spikes in a Cynomolgus Monkey Model of TLE

**Figure 7:**
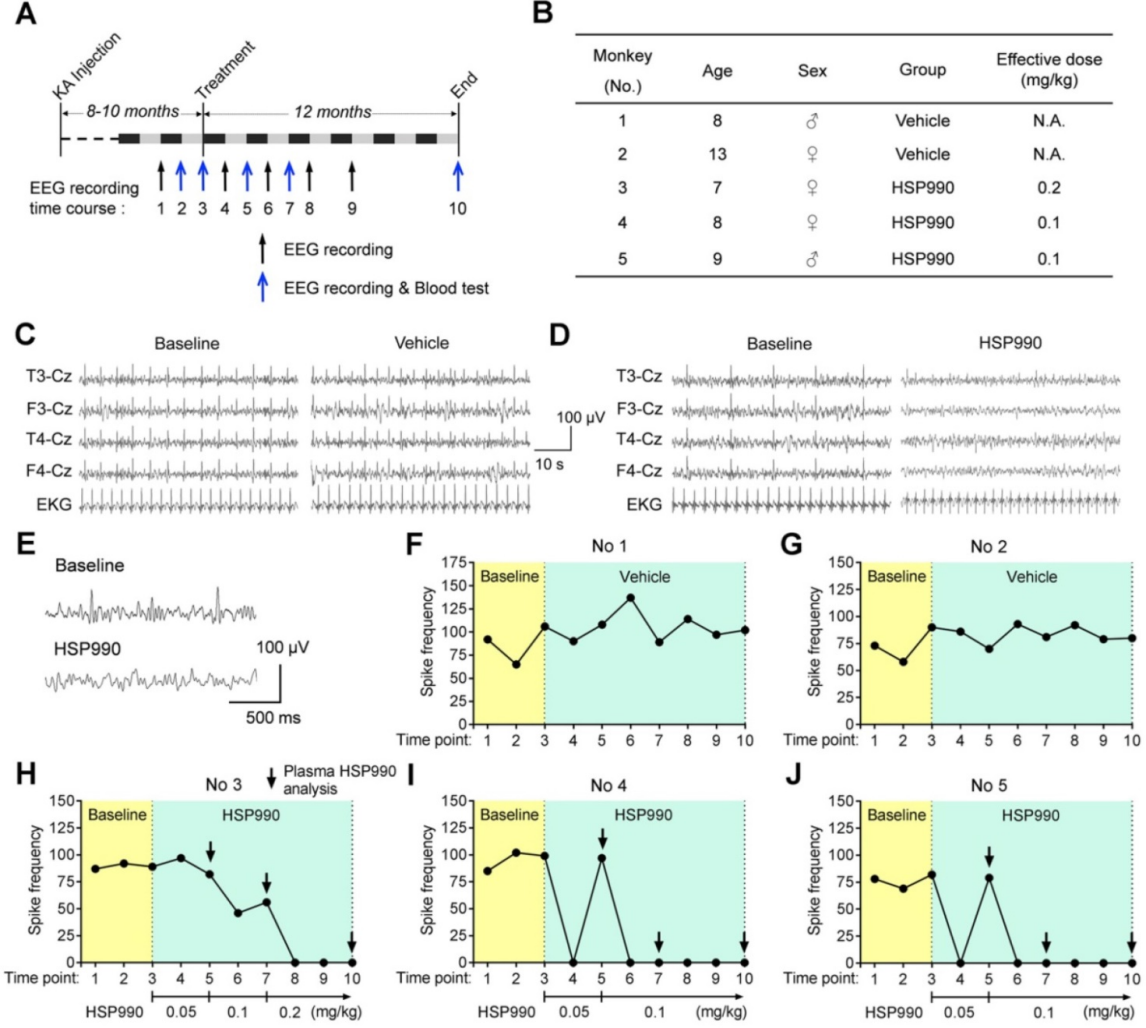
** The suppression of epileptic spikes by HSP990 administration in a cynomolgus monkey model of TLE. (A)** Experimental design. **(B)** Information about the animals used. **(C, D)** Representative EEG traces of 1 min showing EEG activity before and after treatment with vehicle and HSP990, respectively. **(E)** Representative EEG traces showing sharp-and-slow wave complexes during baseline EEG recording and normal EEG activity after HSP990 treatment. **(F-J)** The spike frequencies of baseline (yellow background, 3-time EEG recording) and during treatment with vehicle and HSP990 (green background, 7-time EEG recording), respectively. The dose of HSP990 administration is indicated at the bottom of Figure 7H-J. Arrows in Figure 7H-J indicate the time points when plasma drug concentration was examined in HSP990-treated monkeys. N.A. not available.

Next, we tested the antiepileptic activity of HSP990 through oral administration in a cynomolgus monkey model of TLE 9. As reported before, the intra-hippocampal KA monkey models exhibit no evident clonic or convulsive symptoms in the chronic epilepsy state 9-11; therefore, the change in epileptic spike frequencies was used to evaluate potential antiepileptic activity. The experimental flow chart is shown in Figure 7A. Eight to ten months after the last KA injection, HSP990 and vehicle in hard gelatin capsules were orally administered, respectively, to monkeys grouped throughout the entire experiment. The time points of EEG test and blood analysis are indicated in Figure 7A. Two monkeys in the vehicle group and three in the HSP990 group were included in the present study (No 1 and 2 vehicle; No 3-5 HSP990; Figure 7B). Representative EEG traces are shown in Figure 7C and D. During the first three baseline EEG recordings, monkeys had a relatively stable incidence of epileptic spikes at a frequency of 58 -106 per 30 mins (Figure 7F-J). As expected, the vehicle group showed no reduction in epileptic spike frequencies during the entire experiment (Figure 7FG). Based on the dose conversion formula between animals 14, the starting dose of HSP990 was 0.05 mg/kg, which was orally administered to monkeys No 3-5 once every other day. After 1 month's treatment, monkeys No 4 and 5 demonstrated that the epileptic spikes were completely absent from EEG recordings (EEG time point 4; Figure 7I-J), but appeared again one-month later (EEG time point 5; Figure 7I-J), which suggests drug tolerance. We therefore increased the dose of HSP990 to 0.1 mg/kg and found that the two monkeys became completely free from epileptic spikes in the following five EEG recordings (EEG time points 6-10; Figure 7I-J). However, monkey No 3 did not show suppression of epileptic spikes with the doses of either 0.05 or 0.1 mg/kg treatment (Figure 7H). We finally increased HSP990 to 0.2 mg/kg; this dose was effective, and no epileptic spikes were detected in the next three EEG recordings (EEG time points 8-10; Figure 7H).

### Pharmacokinetics and Safety Analysis

**Figure 8:**
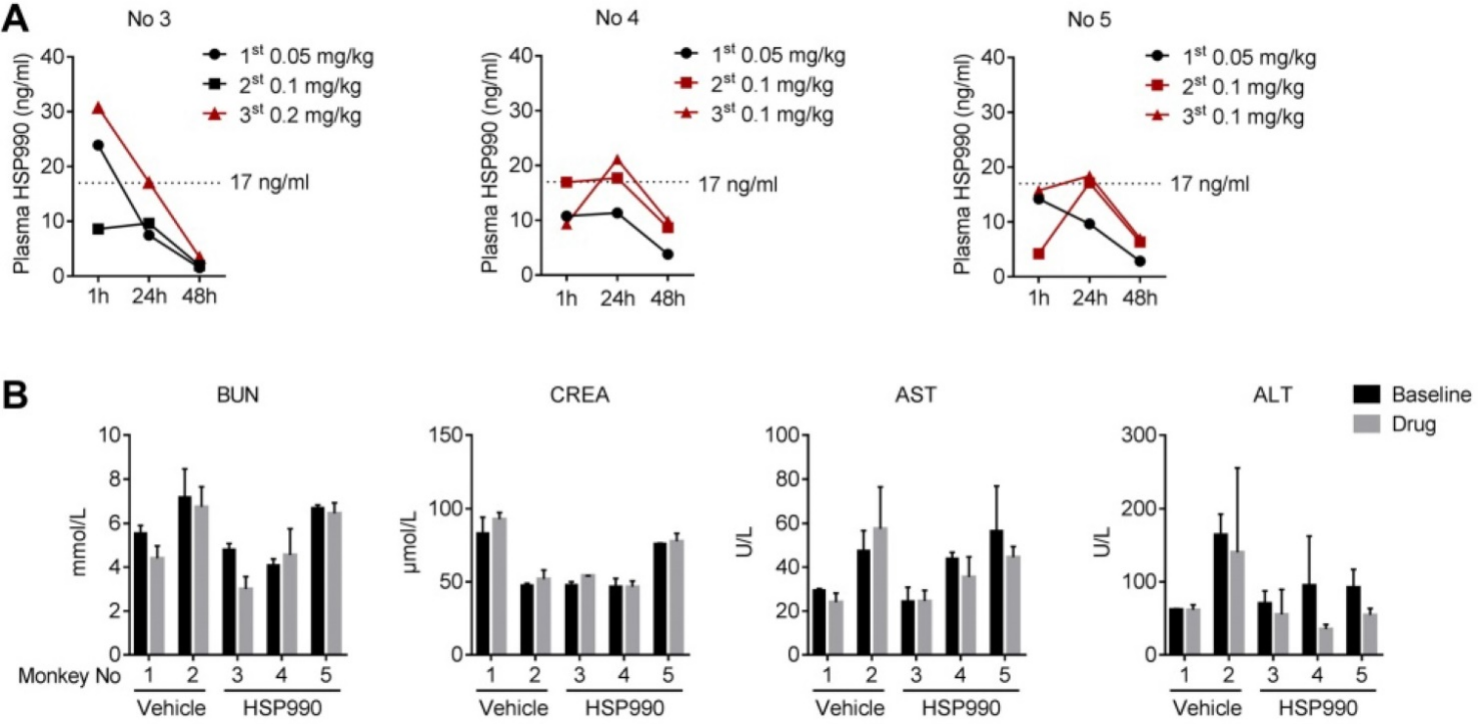
** HSP990 PK analysis and blood biochemical testing. (A)** Plasma HSP990 concentrations at 1 h, 24 h and 48 h of its administration with different doses (3 tests as indicated by arrows in Figure 7H-J). Orange lines indicate simultaneous EEG recording without epileptic spikes.** (B)** The blood levels of BUN, CREA, AST and ALT before and after treatments (n = 2 in baseline levels; n = 3 after treatment; red arrows in Figure 7A indicate analysis time points). AST, aspartate aminotransferase; ALT, alanine aminotransferase; BUN, blood urea nitrogen; CREA, creatinine.

To better understand the effect of HSP990, we analyzed the pharmacokinetics of HSP990 three times at the time points indicated (Figure 7H-J). For each analysis, plasma samples were collected from monkeys at 1, 24, and 48 h of HSP990 administration. As shown in Figure 8A, monkeys No 4 and 5 exhibited a similar PK at different dose levels. Compared with the antiepileptic activity of HSP990, we found that the lowest threshold of effective HSP990 plasma levels was ~17 ng/ml at 24 h of post-dosing when administered with 0.1 mg/kg for monkeys No 4 and 5. Conversely, monkey No 3 exhibited lower plasma HSP990 levels at 24 h of administration than monkeys No 4 and 5, which received the same dose. This demonstrates insufficient inhibition of epileptic spikes (EEG time point 7; Figure 7H). When the dose was increased to 0.2 mg/kg in monkey No 3, the HSP990 plasma level reached 17.2 ng/ml, and the synchronous EEG recording showed complete inhibition of epileptic spikes (EEG time points 8-10; Figure 7H). Collectively, there was a difference in HSP990 pharmacokinetics between individual monkeys, whereas an effective therapeutic response to HSP990 was not observed until its plasma concentration reached 17 ng/ml.

Drug safety was evaluated by examining a series of blood indices and monkey's body weights. Five examinations were performed, two of which were conducted before treatment with either HSP990 or vehicle and three after treatment. Routine blood tests and biochemical analyses showed no signs of hematological toxicity (Table S1 and S2). The levels of AST/ATL and BUN/CREA, whose evaluation represents direct indication of a hepatic or renal injury, were not different before and after treatment in both the vehicle and HSP990 groups (Figure 8B). Moreover, HSP990 had no apparent effect on body weight after 12 months of treatment. Overall, the PK study has identified a potential effective plasma concentration of HSP990 in treating epilepsy, and safety analysis suggested that low-dose HSP990 was unlikely to generate severe adverse effects.

## Discussion

In the present study, we screened and identified HSP990 as a more promising Hsp90 inhibitor for the treatment of TLE. The orally administered HSP990 has greater potency than 17AAG with respect to Hsp90 inhibition, bioavailability and BBB penetration. We demonstrated that low-dose HSP990 exerts antiepileptic activity in mouse and primate models of epilepsy without overt signs of toxicity.

### Properties, Preclinical and Clinical Studies of HSP990

HSP990 is based on a 2-amino-4-methyl-7,8-dihydropyrido[4,3-d]pyrimidin-5(6H)-one scaffold (molecular weight 379.4 g/mol), which is structurally distinct from other Geldanamycin derivatives. HSP990 binds to the N-terminal ATP-binding domain of Hsp90 and exhibits single digit nanomolar IC50 values on Hsp90α and Hsp90β (0.6 and 0.8 nmol/L) 15. In a tumor xenograft model, HSP990 treatment inhibited tumor growth with two different dose schedules, 5 mg/kg twice a week or 15 mg/kg once a week. No significant weight loss or overt signs of toxicity were observed in treated mice 15. In another study, HSP990 was used to treat Huntington's disease (HD). By activating the heat shock response in the brain, the authors demonstrated that a single acute oral dose of 12 mg/kg HSP990 improves huntingtin aggregate load, motor performance and other HD-related phenotypes in the R6/2 mouse model of HD 16. Importantly, the study confirmed the brain-penetrant property of HSP990.

The phase I clinical trial of HSP990 was conducted in 11 patients with advanced solid malignancies, who received 25 mg HSP990 twice a week. Such a dose can be converted into 0.4 mg/kg for a body weight of 60 kg. Although only mild or moderate adverse effects were observed, one patient exhibited dose-limiting neurotoxicities including ataxia, confusion, and visual hallucination 17. Overall, the BBB-penetrant HSP990 exhibits moderate toxicity profiles.

### Low-dose HSP990 is effective in achieving Seizure Control in Mouse models of Epilepsy

The upregulation of Hsp90 plays negative roles in many diseases, but its normal function is to regulate protein stability in normal cells. Accordingly, balancing efficacy and toxicity is essential to achieve a suitable therapeutic index in patients. As an anti-tumor drug, the toxicities of Hsp90 inhibitors are inevitable, as discussed above. Overexpression of Hsp90 has been observed in several types of cancer cells and in astrocytes of the epileptic foci of TLE 18-20. Interestingly, baseline levels of Hsp90 in normal astrocytes are hardly detected. Although increased Hsp90 is involved in epileptogenesis, the present study demonstrated that its expression in reactive astrocytes is still much lower than in neurons. Theoretically, the dose required to completely inhibit astrocytic Hsp90 proteins can suppress only one quarter of Hsp90 proteins in neurons, as determined by the ratio of the Hsp90 protein levels in astrocytes to neurons (Figure 3B). We demonstrated the hypothesis by examining *in situ* Hsp70 because the levels of Hsp70 upregulation represent the degree of Hsp90 inhibition. We found that low-dose HSP990 treatment only induced Hsp70 expression in astrocytes but not in neurons, which indicates that such a treatment dose has minimal effect on neuronal Hsp90. Because the total Hsp90 protein levels in the brain are similar to other tissues, it is reasonable to expect a low-dose systemic drug that can be more safely delivered to achieve Hsp90 inhibition in astrocytes as long as the Hsp90 inhibitor has high BBB penetration.

Although there is no published data reporting the performance of HSP990 on BBB penetration, it can be estimated based on the following information: 1) in the cell-free assay, the IC50 of HSP990 and 17AAG are 0.6 nM and 5 nM, respectively, which suggests that the inhibitory efficacy of HSP990 is ~8-fold higher than that of 17AAG; 2) the present study on normal mice demonstrated that a dose of 0.5 mg/kg HSP990 is required for inducing a peak level of hippocampal EAAT2, which is 40-fold lower than 17AAG, as previously reported 2. Regardless of other influencing factors, the transport efficacy of HSP990 across the BBB should be 5-fold higher than 17AAG. Additionally, with the identical KA mouse model of TLE, we observed a similar antiepileptic activity of HSP990 compared with 17AAG 2. The inhibitory effects of 17AAG and HSP990 on spontaneous seizure activity were 73% and 80%, respectively. Both compounds significantly reduced hippocampal interstitial glutamate levels. In conclusion, low-dose HSP990 exhibits a comparable antiepileptic effect to 17AAG in the KA mouse model of TLE.

### HSP990 modulates the metabolism of Glutamate, Glutamine, Glycine and Serine

After being transported into astrocytes, the glutamate is converted into glutamine by glutamine synthetase (GS), released into the extracellular space, and taken up by neurons 21. The loss of functional EAAT2 could lead to the accumulation of extracellular glutamate, while restoring EAAT2 function did not cause accumulation of neither glutamate nor glutamine, as demonstrated in this study. In fact, both the levels of glutamate and glutamine were significantly reduced. The glutamate levels are directly correlated with the occurrence of epilepsy. Elevated glutamate levels have been reported in human brain tissues and animal models of epilepsy 22. During the seizure period, glutamate levels increased to approximately 10 times their normal values, and then gradually returned to normal when a seizure has ceased 23. Conversely, antiepileptic treatment led to a reduction in brain glutamate levels 24. These findings suggest that reduced levels of glutamate and glutamine may represent a consequence of successful antiepileptic treatment. However, we also observed significantly decrease in serine levels and increase in glycine levels. D-Serine is a coactivator of the NMDA receptors. In hippocampus, D-Serine has a predominant role in driving NMDAR-dependent synaptic plasticity 25. Serine can be converted to glycine by the enzyme serine hydroxymethyltransferase. Glycine regulates hippocampal neuronal activity through GlyR-mediated cross-inhibition of GABAergic inhibition 26. Theoretically, increased glycine levels and decreased serine levels could lead to reduction in nerve excitability. These results imply that the Hsp90 inhibitor HSP990 may affect metabolism of specific amino acids, which induces antiepileptic effects other than enhancing EAAT2 activity. However, how exactly this direct or indirect mechanisms are regulated requires further investigation.

### Drug Efficacy, PK and Safety Study of HSP990 in the Cynomolgus Model of TLE

Non-human primates provide the most accurate models for preclinical studies. In this study, we used the previously established cynomolgus monkey model of TLE to evaluate the antiepileptic activity of HSP990 9. One weakness of the present study is the lack of assessment on epileptic seizures. Unlike the frequently observed convulsive seizures in the KA mouse model, all the intra-hippocampal KA monkey models exhibit no evident clonic or convulsive symptoms in the chronic epilepsy state 9-11, 27. This model mimics simple partial or complex partial seizures with a loss of awareness but few mild motor signs, which are the most frequent seizure types observed in TLE patients. Video recording is not sufficient for identification of epileptic seizures in this TLE monkey model. Long-term, subdermal implantable EEG recording is also difficult in monkeys due to technical challenges. At this stage of the study, the change in epileptic spike frequencies was used to evaluate the antiepileptic activity of HSP990. The presence of epileptic spikes and their frequency and distribution are used to diagnose, classify, and mange some types of epilepsy, including TLE 28. The TLE monkeys exhibited a relatively stable incidence of epileptic spikes in the beginning of drug evaluation study. As frequently occurred in antiepileptic treatment of TLE patients, two HSP990-treated monkeys developed drug tolerance at the dose of 0.05 mg/kg (No 4 and 5), which manifested by loss of drug efficacy during prolonged treatment. This may be attributed to increased drug elimination or functional tolerance. To accurately describe an effective drug concentration, we further evaluated HSP990 levels in plasma. The phase I clinical trial found a high interpatient PK variability of HSP990 17. Consistent with this, monkey No 3 required a 2-fold dose of HSP990 to reach the same plasma concentration compared with monkeys No 4 and 5. The final effective dose of HSP990 in treating the monkey model of TLE is 0.1-0.2 mg/kg, which is still six times lower than the dose used for clinical cancer treatment, as mentioned above (0.4 mg/kg in humans is equivalent to 1.2 mg/kg in monkeys).

Although pathological examination is currently not available for monkeys, blood testing suggests that long-term administration of HSP990 has no overt sign of chronic hepatic and renal toxicity. Some small-molecule Hsp90 inhibitors could induce retinal toxicity 29, but this was not observed in HSP990 administration, as reported in other study 30. In humans receiving HSP990 for cancer therapy, dose-limiting toxicities include central neurological toxicities that comprise myoclonus, fatigue, presyncope, syncope, and decreased appetite 17. Whether the monkeys suffered from these neurological conditions is unclear and needs further investigation.

### The Protective Role of the Hsp90 Inhibitors in AD

Hsp90 can be viewed as a ubiquitous molecular chaperone potentially involved in AD pathogenesis 31-33. A recent study reported that administration of high-dose OS47720 to inhibit Hsp90 in neurons could significantly reverse cognitive deficiency in AD mice by activating Hsp70 and HSF1-mediated synaptic plasticity-related gene expression 34. As mentioned above, the Hsp90 expression in astrocytes is 67% lower than in neurons. Low-dose HSP990 treatment markedly inhibited astrocytic Hsp90 but with minimal effect on neuronal Hsp90, as demonstrated by a specific upregulation of Hsp70 in astrocytes (Figure 3C). Therefore, the improvement in cognitive function in AD mice by low-dose HSP990 seems to be irrelevant to the mechanism reported by Wang et al. Conversely, only a low-dose Hsp90 inhibitor can lead to significant upregulation of EAAT2 levels, whereas a high-dose Hsp90 inhibitor caused transcriptional inhibition of EAAT2 (Figure S2), which raises doubts about whether the protective effects of high-dose Hsp90 inhibitor in AD mice can be attributed to EAAT2.

EAAT2 is lost in the brain of AD patients 35. Takahashi and colleagues found that using LDN/OSU-0212320, an EAAT2 activator, to restore EAAT2 protein and function benefits cognitive functions in the APP/PS1 transgenic mice, but whether this was accompanied by relieved epileptic seizures has not been examined 36. Vossel and colleagues found that 42% of AD individuals without a history of seizures had subclinical epileptiform discharges, and the individuals with epileptiform discharges had a faster rate of cognitive decline 37. A recent study on human patients clinically detected silent hippocampal seizures in an early stage AD 38. Additionally, a study on postmortem brains revealed a positive correlation between EAAT2 expression and cognitive function 39. These observations suggest that the loss of EAAT2 plays critical roles in the cognitive decline of AD. Consistent with this hypothesis, we demonstrated that administration of the Hsp90 inhibitor to increase EAAT2 levels significantly inhibited spontaneous seizures and improved cognitive function in a mouse model of AD. However, we did not observe any change in hippocampal Aβ burden after 4 weeks of HSP990 treatment, even though cognitive function was improved. These results imply that the aberrant neuronal discharges may directly be correlated with cognitive decline in AD, in line with studies on epilepsy reporting that subclinical seizures and spikes can cause significant cognitive impairments 40.

## Conclusions

Excitotoxicity is associated with various neurological disorders, including AD, PD, schizophrenia, epilepsy, and autism 41. While the mechanisms of neurological disorders are not well understood, the dysregulation of EAAT2 may play a significant role in excitotoxicity. The present study demonstrated that a low-dose of HSP990 could prevent EAAT2 loss and play protective roles against excitotoxicity. These results suggest further future investigations on the therapeutic potential of Hsp90 inhibitors in TLE and other excitotoxicity-related disorders.

## Supplementary Material

Supplementary figures and tables.

## Abbreviations

TLE: temporal lobe epilepsy
BBB: blood-brain barrier
PTZ: pentylenetetrazol
KA: kainic acid
SFDs: seizure-free days
IEDs: interictal epileptiform discharges
AD: Alzheimer's disease

## References

[1] Lin CLG, Kong QM, Cuny GD, Glicksman MA (2012). Glutamate transporter EAAT2: a new target for the treatment of neurodegenerative diseases. Future Med Chem.

[2] Sha L, Wang X, Li J, Shi X, Wu L, Shen Y (2017). Pharmacologic inhibition of Hsp90 to prevent GLT-1 degradation as an effective therapy for epilepsy. J Exp Med.

[3] Solit DB, Osman I, Polsky D, Panageas KS, Daud A, Goydos JS (2008). Phase II trial of 17-allylamino-17-demethoxygeldanamycin in patients with metastatic melanoma. Clin Cancer Res.

[4] Egorin MJ, Zuhowski EG, Rosen DM, Sentz DL, Covey JM, Eiseman JL (2001). Plasma pharmacokinetics and tissue distribution of 17-(allylamino)-17-demethoxygeldanamycin (NSC 330507) in CD2F1 mice1. Cancer Chemother Pharmacol.

[5] Wang X, Sha L, Sun N, Shen Y, Xu Q (2017). Deletion of mTOR in Reactive Astrocytes Suppresses Chronic Seizures in a Mouse Model of Temporal Lobe Epilepsy. Mol Neurobiol.

[6] Zeng LH, Ouyang Y, Gazit V, Cirrito JR, Jansen LA, Ess KC (2007). Abnormal glutamate homeostasis and impaired synaptic plasticity and learning in a mouse model of tuberous sclerosis complex. Neurobiol Dis.

[7] Sha LZ, Xing XL, Zhang D, Yao Y, Dou WC, Jin LR (2012). Mapping the spatio-temporal pattern of the mammalian target of rapamycin (mTOR) activation in temporal lobe epilepsy. PLoS One.

[8] Takeda S, Sato N, Uchio-Yamada K, Sawada K, Kunieda T, Takeuchi D (2010). Diabetes-accelerated memory dysfunction via cerebrovascular inflammation and Abeta deposition in an Alzheimer mouse model with diabetes. Proc Natl Acad Sci U S A.

[9] Chen T, Deng Y, Sha L, Shen Y, Xu Q (2019). A cynomolgus monkey model of temporal lobe epilepsy. Brain Res Bull.

[10] Chen N, Liu C, Yan N, Hu W, Zhang JG, Ge Y (2013). A macaque model of mesial temporal lobe epilepsy induced by unilateral intrahippocampal injection of kainic Acid. PLoS One.

[11] Chi Y, Wu B, Guan J, Xiao K, Lu Z, Li X (2017). Establishment of a rhesus monkey model of chronic temporal lobe epilepsy using repetitive unilateral intra-amygdala kainic acid injections. Brain Res Bull.

[12] Marchi N, Granata T, Ghosh C, Janigro D (2012). Blood-brain barrier dysfunction and epilepsy: pathophysiologic role and therapeutic approaches. Epilepsia.

[13] Lin CL, Kong Q, Cuny GD, Glicksman MA (2012). Glutamate transporter EAAT2: a new target for the treatment of neurodegenerative diseases. Future Med Chem.

[14] Nair AB, Jacob S (2016). A simple practice guide for dose conversion between animals and human. J Basic Clin Pharm.

[15] Menezes DL, Taverna P, Jensen MR, Abrams T, Stuart D, Yu GK (2012). The novel oral Hsp90 inhibitor NVP-HSP990 exhibits potent and broad-spectrum antitumor activities *in vitro* and *in vivo*. Mol Cancer Ther.

[16] Labbadia J, Cunliffe H, Weiss A, Katsyuba E, Sathasivam K, Seredenina T (2011). Altered chromatin architecture underlies progressive impairment of the heat shock response in mouse models of Huntington disease. J Clin Invest.

[17] Spreafico A, Delord JP, De Mattos-Arruda L, Berge Y, Rodon J, Cottura E (2015). A first-in-human phase I, dose-escalation, multicentre study of HSP990 administered orally in adult patients with advanced solid malignancies. Br J Cancer.

[18] Wu J, Liu T, Rios Z, Mei Q, Lin X, Cao S (2017). Heat Shock Proteins and Cancer. Trends Pharmacol Sci.

[19] Kandratavicius L, Hallak JE, Carlotti CG Jr, Assirati JA Jr, Leite JP (2014). Hippocampal expression of heat shock proteins in mesial temporal lobe epilepsy with psychiatric comorbidities and their relation to seizure outcome. Epilepsia.

[20] Zheng ZG, Zhang X, Liu XX, Jin XX, Dai L, Cheng HM (2019). Inhibition of HSP90beta Improves Lipid Disorders by Promoting Mature SREBPs Degradation via the Ubiquitin-proteasome System. Theranostics.

[21] Schousboe A, Scafidi S, Bak LK, Waagepetersen HS, McKenna MC (2014). Glutamate Metabolism in the Brain Focusing on Astrocytes. Glutamate and Atp at the Interface of Metabolism and Signaling in the Brain.

[22] Haglid KG, Wang S, Qiner Y, Hamberger A (1994). Excitotoxicity. Experimental correlates to human epilepsy. Mol Neurobiol.

[23] Xiao GH, Xu SW, Song YL, Zhang Y, Li ZY, Gao F (2019). *In situ* detection of neurotransmitters and epileptiform electrophysiology activity in awake mice brains using a nanocomposites modified microelectrode array. Sensors and Actuators B-Chemical.

[24] Melo TM, Nehlig A, Sonnewald U (2006). Neuronal-glial interactions in rats fed a ketogenic diet. Neurochem Int.

[25] Guercio GD, Panizzutti R (2018). Potential and Challenges for the Clinical Use of D-Serine as a Cognitive enhancer. Frontiers in Psychiatry.

[26] Xu TL, Gong N (2010). Glycine and glycine receptor signaling in hippocampal neurons: diversity, function and regulation. Prog Neurobiol.

[27] Lin Z, Meng L, Zou J, Zhou W, Huang X, Xue S (2020). Non-invasive ultrasonic neuromodulation of neuronal excitability for treatment of epilepsy. Theranostics.

[28] Guida M, Iudice A, Bonanni E, Giorgi FS (2015). Effects of antiepileptic drugs on interictal epileptiform discharges in focal epilepsies: an update on current evidence. Expert Rev Neurother.

[29] Kanamaru C, Yamada Y, Hayashi S, Matsushita T, Suda A, Nagayasu M (2014). Retinal toxicity induced by small-molecule Hsp90 inhibitors in beagle dogs. J Toxicol Sci.

[30] Aguila M, Bevilacqua D, McCulley C, Schwarz N, Athanasiou D, Kanuga N (2014). Hsp90 inhibition protects against inherited retinal degeneration. Hum Mol Genet.

[31] Karagoz GE, Duarte AM, Akoury E, Ippel H, Biernat J, Moran Luengo T (2014). Hsp90-Tau complex reveals molecular basis for specificity in chaperone action. Cell.

[32] Schirmer C, Lepvrier E, Duchesne L, Decaux O, Thomas D, Delamarche C (2016). Hsp90 directly interacts, *in vitro*, with amyloid structures and modulates their assembly and disassembly. Biochimica Et Biophysica Acta-General Subjects.

[33] Campanella C, Pace A, Bavisotto CC, Marzullo P, Gammazza AM, Buscemi S (2018). Heat Shock Proteins in Alzheimer's Disease: Role and Targeting. Int J Mol Sci.

[34] Wang B, Liu Y, Huang L, Chen J, Li JJ, Wang R (2017). A CNS-permeable Hsp90 inhibitor rescues synaptic dysfunction and memory loss in APP-overexpressing Alzheimer's mouse model via an HSF1-mediated mechanism. Mol Psychiatry.

[35] Garcia-Esparcia P, Diaz-Lucena D, Ainciburu M, Torrejon-Escribano B, Carmona M, Llorens F (2018). Glutamate Transporter GLT1 Expression in Alzheimer Disease and Dementia With Lewy Bodies. Front Aging Neurosci.

[36] Takahashi K, Kong Q, Lin Y, Stouffer N, Schulte DA, Lai L (2015). Restored glial glutamate transporter EAAT2 function as a potential therapeutic approach for Alzheimer's disease. J Exp Med.

[37] Vossel KA, Ranasinghe KG, Beagle AJ, Mizuiri D, Honma SM, Dowling AF (2016). Incidence and impact of subclinical epileptiform activity in Alzheimer's disease. Ann Neurol.

[38] Lam AD, Deck G, Goldman A, Eskandar EN, Noebels J, Cole AJ (2017). Silent hippocampal seizures and spikes identified by foramen ovale electrodes in Alzheimer's disease. Nat Med.

[39] Kobayashi E, Nakano M, Kubota K, Himuro N, Mizoguchi S, Chikenji T (2018). Activated forms of astrocytes with higher GLT-1 expression are associated with cognitive normal subjects with Alzheimer pathology in human brain. Sci Rep.

[40] Kleen JK, Scott RC, Holmes GL, Roberts DW, Rundle MM, Testorf M (2013). Hippocampal interictal epileptiform activity disrupts cognition in humans. Neurology.

[41] Pajarillo E, Rizor A, Lee J, Aschner M, Lee E (2019). The role of astrocytic glutamate transporters GLT-1 and GLAST in neurological disorders: Potential targets for neurotherapeutics. Neuropharmacology.

