# Supplementary material for: Hsp90 inhibitor HSP990 in very low dose upregulates EAAT2 and exerts potent antiepileptic activity
Source: Theranostics. 2020 Jul 9;10(18):8415–29. doi: 10.7150/thno.44721 (PMC7381737; doi:10.7150/thno.44721)
Supplement: Supplementary file 1 — Supplementary figures and tables. [file thnov10p8415s1.pdf]

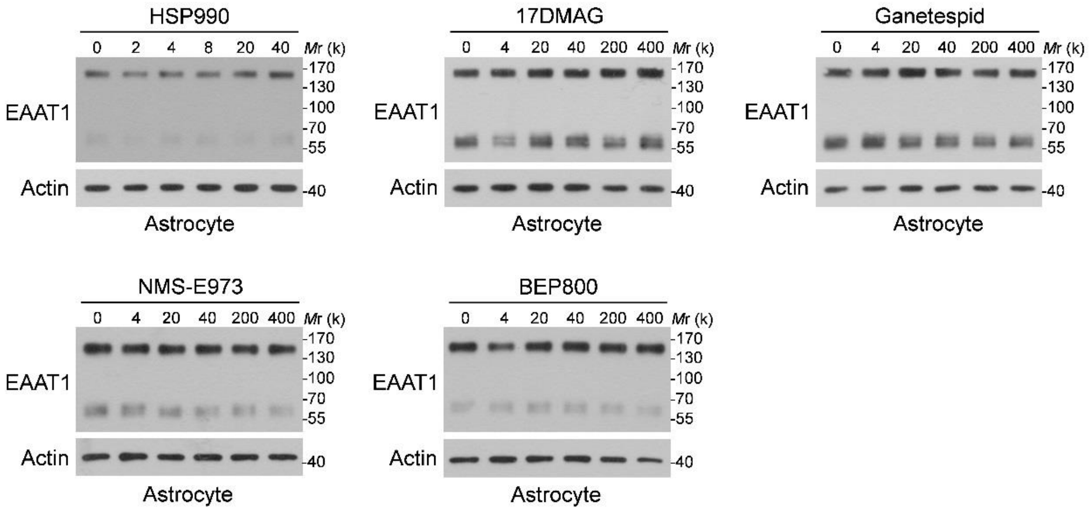

Supplementary Figure 1. Western blot analysis of EAAT1 in astrocytes treated with the 5 Hsp90 inhibitors for 24 h (n = 3 for each group).

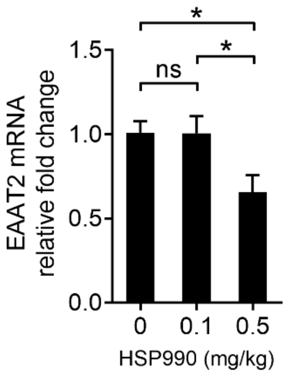

Supplementary Figure 2. Expression of *EAAT2* mRNA levels in the hippocampus ispileteral to KA injection of mice with treatment of HSP990 (0, 0.1, 0.5 mg/kg, administered for 3 times in a week).

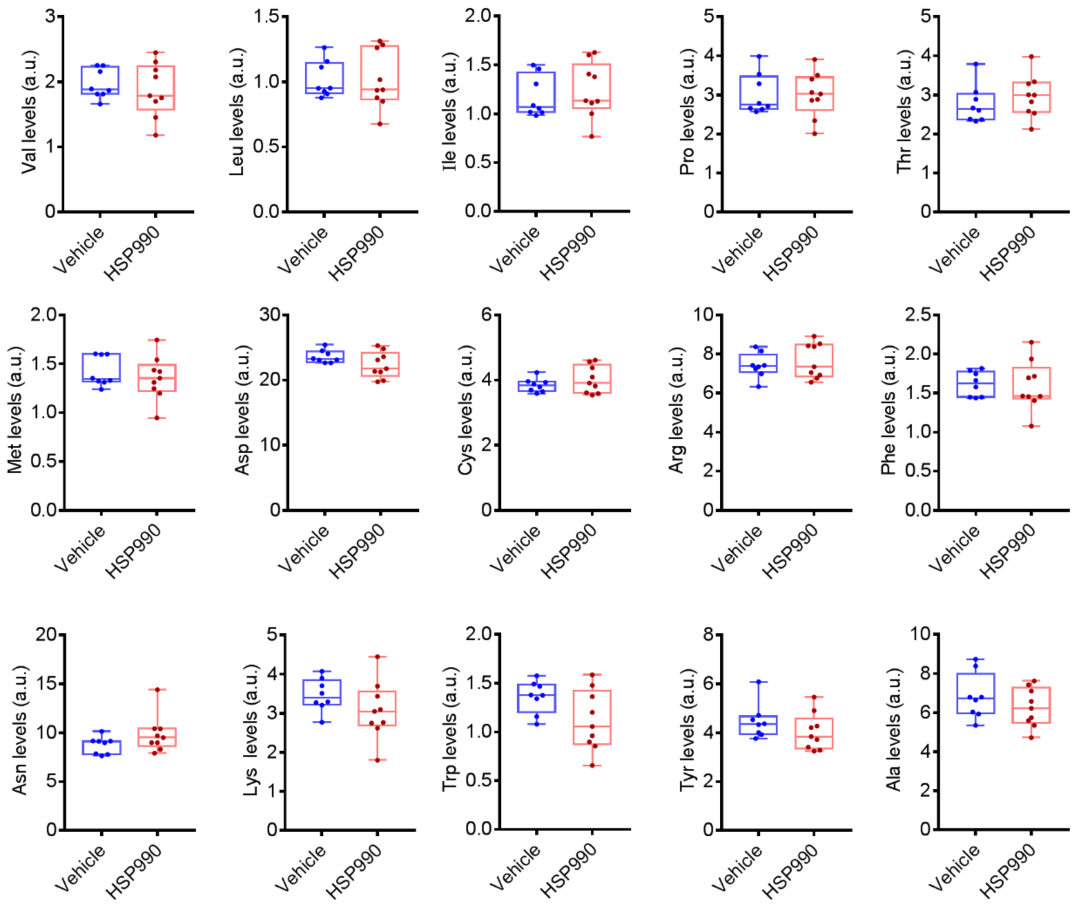

Supplementary Figure 3. Metabolomic profiles of the 15 amino acids levels in the hippocampus from vehicle- and HSP990-treated epileptic mice. Student's t-test failed to show a significant difference between all groups (n = 8-9 each group). a.u., arbitrary units.

Supplement Table 1. Routine blood tests for monkeys treated with HSP990 or vehicle.

Monkey ID		Test (No.)	Weight (kg)	wbc	rbc	hb	hct	mcv	mch	mchc	rdw	plt	mpv	ne#	ne%	ly#	ly%	eo#	eo%	mo#	mo%	ba#	ba%
Vehicle	1	1	9.30	11.16	5.95	177	40.9	68.7	29.7	433	14.2	302.	14.5	5.63	50.49	4.51	40.37	0.54	4.81	0.47	4.22	0.01	0.11
		2	9.30	14.16	6.32	198	47.1	74.5	31.3	420	13.6	346.	14.8	10.19	71.98	2.05	14.51	0.85	6.02	1.03	7.27	0.03	0.22
		3	9.30	7.22	5.44	165	37.7	69.3	30.3	438	14.5	271.	12.8	3.51	48.62	3.32	45.95	0.27	3.68	0.13	1.76	0.00	0.00
		4	9.30	12.90	5.83	168	40.0	68.6	28.8	420	14.7	332.	13.2	8.26	64.03	3.99	30.92	0.38	2.95	0.25	1.94	0.02	0.16
		5	10.0	11.58	5.66	168	39.1	69.1	29.7	430	14.6	281.	12.2	6.64	57.37	4.37	37.73	0.22	1.91	0.32	2.79	0.02	0.19
	2	1	3.84	11.10	6.73	174	39.6	58.8	25.9	439	15.4	392.	10.6	3.78	34.03	6.20	55.85	0.54	4.83	0.58	5.25	0.00	0.04
		2	3.84	9.16	6.46	181	41.2	63.8	28.0	439	14.4	411.	10.4	3.13	34.20	4.97	54.22	0.46	5.00	0.60	6.58	0.00	0.00
		3	3.80	10.06	5.10	140	31.1	60.9	27.5	450	14.9	349.	10.6	3.29	32.75	6.28	62.44	0.28	2.75	0.20	2.00	0.01	0.07
		4	3.70	9.80	6.25	155	37.0	59.2	24.8	419	14.9	404.	9.9	4.29	43.81	4.70	47.92	0.44	4.54	0.36	3.67	0.01	0.06
		5	3.80	12.36	6.33	171	37.2	58.8	27.0	460	16.3	347.	9.8	5.11	41.31	6.63	53.66	0.24	1.96	0.37	2.97	0.01	0.09
HSP990	3	1	4.44	11.16	6.35	175	39.3	61.9	27.6	445	16.3	283.	11.5	4.07	36.44	5.85	52.39	0.42	3.77	0.82	7.32	0.01	0.10
		2	4.44	11.84	5.76	179	40.9	71.0	31.1	438	14.6	263.	13.6	4.66	39.40	5.48	46.25	0.79	6.64	0.89	7.48	0.03	0.25
		3	4.46	8.10	5.90	167	38.8	65.7	28.3	430	14.8	282.	10.6	2.34	28.95	5.16	63.67	0.32	3.90	0.28	3.43	0.00	0.04
		4	4.60	10.16	5.63	156	36.1	64.2	27.7	432	15.3	395.	10.3	5.60	55.07	4.08	40.14	0.26	2.57	0.23	2.22	0.00	0.00
		5	4.80	8.32	6.33	185	40.2	63.5	29.2	460	16.1	395.	11.9	4.52	54.30	3.48	41.79	0.18	2.18	0.13	1.56	0.01	0.16
	4	1	3.90	7.36	6.21	184	38.8	62.5	29.6	474	16.8	281.	10.4	3.54	48.08	3.13	42.48	0.36	4.90	0.33	4.50	0.00	0.04
		2	3.90	12.44	6.46	200	44.9	69.5	31.0	445	14.7	274.	11.8	8.13	65.38	2.82	22.69	0.54	4.36	0.89	7.15	0.05	0.42
		3	3.80	6.28	5.59	157	36.4	65.2	28.1	431	16.4	288.	9.0	1.92	30.50	3.88	61.78	0.26	4.16	0.22	3.50	0.00	0.05
		4	3.70	11.14	5.59	158	36.0	64.4	28.3	439	15.3	264.	10.5	4.16	37.34	5.66	50.80	0.74	6.66	0.57	5.14	0.01	0.06
		5	3.80	7.36	6.32	178	39.8	63.0	28.2	447	16.5	317.	9.6	3.39	46.07	3.38	45.89	0.32	4.36	0.26	3.59	0.01	0.08
	5	1	6.32	13.18	6.46	181	44.1	68.2	28.0	410	14.6	135.	13.4	4.21	31.94	7.77	58.99	0.50	3.76	0.66	5.04	0.04	0.27
		2	6.32	17.86	6.00	200↑	44.0	73.3	33.3	455	14.4	194.	16.3	10.60	59.33	5.41	30.27	0.80	4.49	0.97	5.45	0.08	0.46
		3	6.32	13.96	5.45	170	38.2	70.1	31.2	445	14.3	191.	11.6	8.02	57.48	5.13	36.74	0.38	2.75	0.37	2.67	0.05	0.36
		4	6.30	14.54	5.53	166	38.2	69.0	30.0	435	14.1	278.	13.6	8.91	61.30	4.63	31.81	0.54	3.74	0.43	2.96	0.03	0.19
		5	6.20	8.52	5.64	160	37.5	66.5	28.4	427	15.0	258.	12.9	3.98	46.69	4.12	48.36	0.23	2.69	0.18	2.15	0.01	0.10
Normalized Value				5.0-21.1	4.6-7.3	87-192	30.3-47.3	69.5-83.4	19.6-34.0	27.1-33.0	12.0-27.0	259-734	5.0-15.0										

wbc: White blood cell; rbc: Red blood cell; hb: Hemoglobin; hct: Packcd cell volume; mcv: Mean Corpuscular Volume ; mch: Mean Corpuscular hemoglobin; mchc: Mean corpusular hemoglobin concerntration; rdw: Red Cell volume Distribution Width; plt: Blood platelet; mpv: Mean Platelet Volum; ne: Neutrophil; ly: Lymphocyte

eo: Eosinophil; mo: Monocytes; ba: Basophil

Supplement Table 2. Biochemical analyses for monkeys treated with HSP990 or vehicle.

Monkey ID		Test (No.)	Weight (kg)	Hepatic function index									Renal function index				Blood fat index			
				ALT	AST	AST/ALT	ALP	TBIL	TP	ALB	GLO	ALB/GLO	BUN	CREA	CK	GLU	CHOL	TG	HDL	LDL
Vehicle	1	1	9.3	61.40	30.00	0.5	238	32.2	77.5	42.7	34.8	1.2	5.8	75.5	115.8	3.7	2.52	0.65	1.32	1.25
		2	9.3	62.80	29	0.5	346	46.9	84	47.4	36.6	1.3	5.3	91	119.2	5.6	2.73	0.58	1.48	1.36
		3	9.3	66.6	21	0.3	263	30.7	71.1	42.1	29	1.5	4	96.1	NA	3.9	2.32	0.56	1.15	1.21
		4	9.3	56.8	27	0.5	238	NA	80.5	46.2	34.3	1.3	4.8	89.5	115.4	5.3	2.4	0.51	NA	NA
		5	10	81.5	43	0.5	116	4.8	76.7	43.3	33.4	1.3	5.1	107.3	411.1	3.9	2.26	0.44	1.17	0.84
	2	1	3.84	144.1	41	0.3	82	38.3	63.2	35.5	27.7	1.3	8.1	48.6	130.3	7.4	3.06	1.21	1.52	1.5
		2	3.84	184.3	54	0.3	95	59.1	68	38	30	1.3	6.3	46.8	118.9	5	3.08	1.66	1.45	1.52
		3	3.8	221.8	71	0.3	127	32.6	60.1	32.2	27.9	1.2	6.1	56.2	NA	4.4	2.39	1.45	1.1	1.27
		4	3.7	58.9	44	0.7	227	43.9	66.7	33.9	32.8	1	7.4	47.4	81.2	8	2.8	0.74	1.25	1.5
		5	3.8	120.4	53	0.4	66	4.5	65.7	34.7	31.0	1.1	7.9	68.6	207.0	3.6	3.11	1.96	1.20	1.37
HSP990	3	1	4.44	82.5	29	0.4	141	27.4	75.7	42.1	33.6	1.3	4.6	49.4	182.7	4.9	3.08	1.06	1.4	1.67
		2	4.44	58.2	20	0.3	169	26.9	66.7	39	27.7	1.4	5	46.4	120.1	6.3	2.54	0.79	1.37	1.27
		3	4.46	79.6	28	0.4	174	42.2	68.1	40.9	27.2	1.5	2.6	54.2	NA	4.9	2.41	0.7	1.14	1.26
		4	4.6	30.9	21	0.7	167	30.1	68.4	42.3	26.1	1.6	3.4	54.1	91.2	4.3	2.27	0.63	1.21	1.05
		5	4.8	49.8	33	0.7	190	8.3	75.9	42.8	33.1	1.3	3.3	70.1	196.0	4.8	2.89	0.67	1.42	1.20
	4	1	3.9	142.8	46	0.3	181	33.5	66.3	37.8	28.5	1.3	4.3	43.2	132.5	3.8	3.93	0.58	2.04	1.84
		2	3.9	47.3	42	0.9	263	28.1	71.2	40.6	30.6	1.3	3.9	50.7	151.9	5	4.08	0.46	2.2	1.96
		3	3.8	39.8	29	0.7	194	17.2	66.2	38.7	27.5	1.4	3.7	49.4	NA	3.5	4.36	0.69	2.44	1.93
		4	3.7	30.7	42	1.4	218	24.6	69.6	39.6	30	1.3	5.4	43.8	104	5	4.38	0.49	2.29	1.93
		5	3.8	54.2	49	0.9	217	2.8	75.2	40.2	35.0	1.1	4.1	52.9	98.3	3.8	5.08	0.65	2.70	1.76
	5	1	6.32	109.8	71	0.6	150	36.2	68.7	42.3	26.4	1.6	6.6	75.7	292.8	4	3.03	0.67	1.83	1.26
		2	6.32	74.2	42	0.6	154	27.5	67.3	37.9	29.4	1.3	6.8	76.3	123.7	5.1	2.94	1.05	1.53	1.4
		3	6.32	61	41	0.7	137	39.4	65.2	39.8	25.4	1.6	6.1	81.6	NA	4.1	2.84	1.04	1.39	1.42

		4	6.3	48.1	48	1	140	49	66.3	40.2	26.1	1.5	6.8	73.9	110	4.1	3.1	1.41	1.44	1.46
		5	6.2	46.6	51	1.1	137	2.0	67.1	39.4	27.7	1.4	7.0	104.7	222.4	3.2	3.22	0.98	1.59	1.39
**Normalized Value**				**30-46**	**29-43**	**0.63-1.43**	**151-205**	**22.4-34.2**	**64-82**	**35.8-48.4**	**20-45**	**1.2-2.3**	**2.5-6.5**	**42.5-92.3**	**179-257**	**3.8-6.1**	**3.60-4.68**	**0.56-1.71**	**0.78-2**	**0-3.7**

ALT: Alanine aminotransferase; AST: Aspartate aminotransferase; ALP: alkaline phosphatase; TBIL: total bilirubin; TP: Total Protein; ALB: albumin; GLO: Globulin; BUN: blood urea nitrogen; CK: Creatine Kinase; GLU: Blood glucose; CHOL: Cholesterol; TG: Trilaurin; HDL: high density lipoproteins; LDL: low density lipoproteins
